# Registries supporting new drug applications

**DOI:** 10.1002/pds.4332

**Published:** 2017-10-06

**Authors:** Carla J. Jonker, H. Marijke van den Berg, Marcel S.G. Kwa, Arno W. Hoes, Peter G.M. Mol

**Affiliations:** ^1^ Dutch Medicines Evaluation Board (CBG‐MEB) Utrecht The Netherlands; ^2^ Julius Center for Health Sciences and Primary Care University Medical Center Utrecht Utrecht The Netherlands; ^3^ Department of Clinical Pharmacy and Pharmacology University Medical Center Groningen Groningen The Netherlands

**Keywords:** disease registry, drug registry, innovative drugs, new drugs, orphan drugs, pregnancy registry

## Abstract

**Purpose:**

Knowledge of the benefits and risks of new drugs is incomplete at the time of marketing approval. Registries offer the possibility for additional, post‐approval, data collection. For all new drugs, which were approved in the European Union between 2007 and 2010, we reviewed the frequency, the type, and the reason for requiring a registry.

**Methods:**

The European Public Assessment Reports, published on the website of the European Medicine Agency, were reviewed for drugs approved by the Committee for Medicinal Products for Human Use. We searched for key characteristics of these drugs, including therapeutic area (ATC1 level), level of innovation (the score is an algorithm based on availability of treatment and therapeutic effect), and procedural characteristics. In addition, we identified if these registries were defined by disease (disease registry) or exposure to a single drug (drug registry).

**Results:**

Out of 116 new drugs approved in the predefined period, for 43 (37%), 1 to 6 registry studies were identified, with a total of 73 registries. Of these 46 were disease registries and 27 (single) drug registries. For 9 drugs, the registry was a specific obligation imposed by the regulators. The level of innovation and the orphan status of the drugs were determinants positively predicting post‐approval registries (OR 10.3 [95% CI 1.0‐103.9] and OR 2.8 [95% CI 1.0‐7.5], respectively).

**Conclusions:**

The majority of registries required by regulators are existing disease registries. Registries are an important and frequently used tool for post‐approval data collection for orphan and innovative drugs.

## 1 INTRODUCTION

Evidence regarding benefit and especially risks of drugs is still limited by the time they are approved by regulatory agencies. Therefore, regulators require additional evidence regarding safety and real‐world effectiveness throughout the remainder of the drug's life cycle.^1^ In some situations, companies are required to provide data from randomized controlled trials in order to establish remaining uncertainties about the benefits and risks of new drugs. Once approved, the number of patients exposed to the drug will be much larger, long‐term data will become available, and safety concerns that could not be detected during clinical trials may be identified. Hence, data collected post‐authorization are critical for learning more about the benefit‐risk balance of new drugs. The Food and Drug Administration (FDA) in the USA and the European Medicine Agency (EMA) in Europe have developed extensive guidance for industry indicating how to address identified and potential safety concerns and how to deal with missing data.^2^, ^3^ These pharmacovigilance activities focus on monitoring real‐life clinical use, including the systematic collection of observational data in registries. Data collected post‐approval through these registries can be used to complement pre‐registration study data to address existing knowledge gaps, eg, missing data regarding children, use during pregnancy, and effects of long‐term treatment. A registry can be used as a data source for other studies, such as studies to measure the effectiveness of risk minimization measures and drug utilization studies.^3^

In Europe, the Committee for Medicinal Products for Human Use (CHMP) is responsible for the scientific evaluation and approval of drugs for use within the European Union. Increasingly more drugs have been approved based on limited data sets during the last decade; eg, 30 drugs were conditionally approved between 2006 and June 2016.^4^ Earlier, we have shown that this trend has not necessarily lead to more safety issues.^5^ For many of these drugs, registries have been proposed to fill the knowledge gap.

Although registries are suggested and approved as a tool for post‐approval collection of additional data for new drugs, it is currently unknown how often this tool is being used, for how many and what type of drugs, and what the rationale is to for requesting a registry. Therefore, the goal of this study was to assess the frequency and the reasons for requesting post‐approval registries in Europe and to examine the type of registries (drug or disease). Further, we investigated whether registries had been imposed by the regulatory authority as a specific obligation or had been “spontaneously” promised by a company in order to address remaining uncertainties on drug benefits and risks. We examined the rationale (eg, safety concerns or long‐term efficacy) underlying the decision to set up a registry. Additionally, we explored what drug characteristics (eg, ATC‐code, level of innovation and size of pre‐approval safety population) and procedure‐related determinants (eg, type of procedure or the existence of an orphan status) predicted a post‐marketing registry to be included in a drug dossier.

**KEY POINTS**

- One‐third of all drugs approved in Europe, between 2007 and 2010, were coupled with a requirement for a registry, mainly with the purpose of providing additional data because of safety concerns.
- The majority of these registries are existing disease registries.
- For orphan and innovative drugs, registries are an important tool for post‐approval data collection.

## 2 METHODS

We performed a retrospective review of drugs approved by the CHMP in the European Union.

### 2.1 Data source

We identified drugs that were approved by the CHMP between January 1, 2007 and December 31, 2010 from the European Commission's Community Register (http://ec.europa.eu/health/documents/community‐register/html/index_en.htm). Only drugs approved on the basis of a full application dossier for a new active substance and biosimilars were included in the dataset. The date of approval is defined as the date of publication of the European Decision.

### 2.2 Primary outcome

The aim of the study was to investigate the frequency and reason for a requirement for a post‐approval registry study to complement the marketing authorization dossier of new drugs. Scientific and regulatory information was collected from the European Public Assessment Reports (EPARs), which are accessible through the EMA website (www.ema.europe.eu). The requirement to set up a registry was identified from the Risk Management Plan (RMP) summary of the EPAR. In this summary safety specifications, proposed pharmacovigilance and risk minimization activities are recorded. We included all registries that were mentioned in the EPAR. A registry is defined as an organized system that uses observational methods to collect uniform data on specified outcomes in a population defined by a particular disease, condition, or exposure.^3^ We excluded studies with a single research question collecting data from 1 or more electronic health records database. In line with Bouvy et al., we also excluded non‐interventional, open‐label, prospective short‐term observational studies (2 years or less).^6^ These studies were considered to be designed for a specific research question rather than a long‐term study in a registry where routine clinical data are collected systematically. Both registries recorded as a specific or imposed obligation conform to annex II of the Marketing Authorization, and those required to investigate a safety concern are included. If more details were needed or if the information in the EPAR was not conclusive, data were obtained from the RMPs and study reports, which were retrieved from the database available at the Medicine Evaluation Board. Data were extracted by CJ; all data were systematically checked by PM or MK to ensure accuracy of extracted information. Any discrepancies were resolved in discussion with CJ, MK, and PM.

### 2.3 Characteristics of registries, drugs, and procedures

We retrieved a number of relevant characteristics of the identified registries. First, we identified in the dossier the primary goal for requiring the registry, eg, to address safety, effectiveness, or pregnancy outcomes.

Second, we ascertained whether the specified outcome was defined by the disease (disease registry) or exposure to a single product or drug (drug registry). Drug registries could also include a class of drugs, but in our data set, only single‐drug registries were identified.

To identify determinants for requiring a post‐approval registry, we identified characteristics related to the nature of drug and the nature of the procedure that we hypothesized could influence the decision to require a registry. First, the therapeutic area was classified using the anatomical main group of the Anatomical Therapeutic and Chemical code (ATC‐1 level, http://www.whocc.no/atc_ddd_index). Second, the type of molecule was categorized as either a small molecule, a vaccine, or a biosimilar, in accordance with the European legal definitions.^7^ Third, we classified the level of innovation of a new drug using an algorithm developed by Motola et al.^8^ Drugs were classified based on a sequential assessment of the availability of alternative treatment options for a particular disease and the therapeutic effect they had demonstrated in clinical studies, both as assessed at the time of approval. The algorithm graded drugs based on these considerations as (A) important, (B) moderate, or (C) modest innovations or as “mere” pharmacological/technological innovations.^9^ Consequently, drugs classified as important innovations target diseases where treatment is not available and have demonstrated major benefits on clinical endpoints or established surrogate parameters.^10^

Fourth, we determined the size of the safety population; the total number of subjects exposed to the drug for any duration in the clinical development program before approval. Finally, 2 procedural characteristics were identified—orphan drug and registration type (standard, under exceptional circumstances or receiving conditional approval) as defined in the Notes to Applicant.^9^

### 2.4 Statistical analyses

Univariate and multivariate logistic regressions were applied to identify, which key drug and procedural characteristics were independent determinants of the requirement for a post‐approval registry. Characteristics that were potentially associated with inclusion of a registry in the dossier (*p* < 0.1) were included in the multivariate model. In the final model, only characteristics reaching a significant level of *p* < 0.05 were considered as statistically significantly associated with the primary outcome.

## 3 RESULTS

Between January 1, 2007 and 31 December 31, 2010, 116 new drugs (new active substances and biosimilars) were approved in Europe by the CHMP. A total of 73 registries were included in the RMPs of 43 (37%) of these newly approved products. For 29 of these new drugs, there was a post‐approval requirement for a single registry, and for 14 drugs, there was a requirement for between 2 and 6 registries (Table 1), implying that for 73 new drugs, there was no need for a registry. For only 9 drugs, registries were imposed by the CHMP. For drugs subjected to a registry, the size of safety population ranged between 94 and 13 000 patients; 15 drugs had an orphan status, and 13 drugs were approved under exceptional circumstances or were conditionally approved (Table 2 and Supporting Information Appendix S1 for individual drugs).

**Table 1:** Key characteristics of 73 registries

	All registries, *N* (%)
Total	73 (100%)
Primary goal	
Safety	39
Safety and effectiveness	7
Pregnancy	27
Disease	46 (63%)
Drug	27 (37%)
Number of registries per drug	
None	73
One	29
Two	6
Three	4
More than 4	6
Registry imposed	
Yes	9 (12%)
No	64 (88%)

**Table 2:** Key characteristics of new drugs approved^a^ with and without registries 2007 to 2010

	All drugs, *N* (%)	Registry,^b^ *N* (%)		Univariate	Multivariate*
		Yes	No	OR (95% CI)	OR (95% CI)
Total	116 (100)	43 (37)	73 (63)		
Drug characteristics					
Therapeutic area (ATC 1 level)					
A	12 (100)	5 (42)	7 (58)	1.9 (0.5;7.5)	
B	12 (100)	3 (25)	9 (75)	0.9 (0.2;4.0)	
J	26 (100)	12 (46)	14 (54)	2.3 (0.8;6.7)	
L	29 (100)	13 (45)	16 (55)	2.2 (0.8;6.2)	
Other^c^	37 (100)	10 (27)	27 (73)	Ref	
Type of molecule					
Biological	30 (100)	15 (50)	15 (50)	1.5 (0.4;5.3)	
Small molecule	71 (100)	22 (31)	49 (69)	0.7 (0.2;2.1)	
Vaccine	15 (100)	6 (40)	9 (60)	Ref	
Level of innovation^d^					
A: Important	7 (100)	6 (86)	1 (14)	**16.0 (1.7;147.1)**	**10.3 (1.0;103.9)**
B: Moderate	42 (100)	18 (43)	24 (57)	2.0 (0.8;4.9)	1.2 (0.4;3.5)
C: Modest	23 (100)	7 (30)	16 (70)	1.2 (0.4;3.6)	0.8 (0.2;2.6)
Pharm/tech	44 (100)	12 (27)	32 (73)	Ref	Ref
Size of safety population^e^					
Median (range)	1549 (94‐13 000)	1002 (94‐13 000)	1811 (119‐10 257)	1.0 (1.0;1.0); *p* = 0.11	
Procedural characteristics					
Orphan medicinal drug^f^ (yes)	26 (100)	15 (58)	11 (42)	**3.0 (1.2;7.4)**	**2.8 (1.0;7.5)**
CA^g^ and EC^h^ registration (yes)	23 (100)	13 (57)	10 (43)	**2.7 (1.1;6.9**)	1.7 (0.6;5.0)

*p* < 0.05 in bold type face.

* All determinants with *p* < 0.1 were included in the multivariate analyses.

a Date of approval is date of publication of European Decision.

b A registry was promised in the European Public Assessment Report (EPAR, as part of the RMP).

c Therapeutic area classified using the anatomical main group of the Anatomical Therapeutic and Chemical Code. All drugs that are not classified as A (alimentary tract and metabolism), B (blood and blood forming organs), J (anti‐infectives for systemic use), or L (antineoplastic and immunomodulating agents) are classified as other.

d The drug is an important, moderate, modest of pharmacological, or technological innovation.

e Size of safety population is the number of patients that have been analyzed in the safety analysis (initial application, in EPAR).

f The drug has an orphan status.

g The drug was given a conditional approval (CA).

h The drug is approved under exceptional circumstances (EC).

The primary goal of 39 of the 73 registries was to collect safety outcomes; in 7 cases, it was to collect safety outcome and real‐world effectiveness data; and in 27 cases, it was to collect data on potential birth defects when the drug was taken during pregnancy. The most common aims of these registries were to increase knowledge on identified and potential risks or information that was missing—especially pregnancy outcome—at the time of approval.

We identified 27 (37%) drug registries that were set up by companies to monitor use and outcomes of their drug specifically. Only patients using these specific drugs are enrolled into these registries. The use and outcomes of treatment with a drug is monitored in 46 (63%) disease registries, in which patients will be enrolled with a specific diagnosis or disease, irrespective of the drug(s) they are using. Examples of disease registries are the Swedish and German rheumatology registries Antirheumatic Therapies in Sweden (ARTIS) and Rheumatoid Arthritis Observation of Biologic Therapy (RABBIT), in which safety data are collected in patients with rheumatoid arthritis for the recently approved drugs abatacept, certolizumab, golimumab, and tocilizumab.^11^ Similarly, for 3 filgrastim biosimilars, safety and immunogenicity are collected in the Severe Chronic Neutropenia (SCN) European registry. The SCN registry monitors clinical progress and treatment and adverse events for patients with SCN, regardless of their therapy.^12^

A specific kind of registry is the pregnancy registry. Of the 27 identified pregnancy registries, 11 were set up specifically to monitor the impact on offspring of a specific drug taken during pregnancy. In the remaining 16 cases, data were collected from existing pregnancy registries; eg, for darunavir, etravirine, maraviroc, raltegravir, and telbivudine, pregnancy outcome data are collected from the Antiretroviral Pregnancy Registry. This is an existing pregnancy registry, set up in 1989 for pregnant women who are exposed to antiretroviral drugs, intending to generate early signals of teratogenic effects associated with prenatal exposure to antiretroviral products.^13^ The registry enrolls human immunodeficiency virus infected patients through their healthcare providers (http://www.apregistry.com/).

We identified registries that were imposed by the CHMP for only 9 drugs, suggesting that registries are specific measures taken in the framework of the marketing authorization. Six of these drugs (amifampridine, canakinumab, idursulfase, mecasermin, rilonacept, and tocofersolan) were approved under exceptional circumstances, because at the time of approval no comprehensive data on the safety and efficacy under normal conditions of use could be provided. Two drugs (both pandemic influenza vaccines) had received a conditional approval, which means that the company will be required to provide confirmative data in a short timeframe, and 1 drug (lenalidomide) had a regular approval.

Four of the imposed registries were set up with the aim to collect safety and real‐world effectiveness data: amifampridine (symptomatic treatment of adults with Lambert‐Eaton myasthenic syndrome); canakinumab, rilonacept (both for the treatment of patients with severely symptomatic cryopyrin‐associated periodic syndromes (CAPS)); and idursulfase (for the treatment of patients with Hunter syndrome). Three registry studies set up for, respectively, mecasermin (treatment of growth failure in children and adolescents with severe primary insulin‐like growth factor‐1 deficiency), lenalidomide (for the treatment of multiple myeloma), and tocofersolan (vitamin E deficiency due to digestive malabsorption in pediatric patients with congenital chronic cholestasis or hereditary chronic cholestasis) focused on the collection of safety data.

Pregnancy registries were imposed for 2 (adjuvanted) pandemic influenza vaccines. Safety during pregnancy (eg, risk of birth defects) was unknown at the time of marketing approval, because of the lack of evidence in pregnant women. The regulatory authorities designated the lack of a pre‐registration data as important missing information, considering that pregnant women are an important target population for these vaccines as influenza is likely to cause more severe illness in pregnant women.^14^ It is noteworthy, though, that the applications for the pandemic influenza vaccines and rilonacept are now withdrawn in the European Union, all 3 for commercial reasons.

### 3.1 Determinants for registries

We explored if specific drug or procedural characteristics were associated with whether a registry was imposed by the regulatory authority or the initiative of the applicant. We used logistic regression to examine this issue. In the univariate analysis, level of innovation (important innovation OR 16.0 [95% CI 1.7‐147.1]), orphan drug (OR 3.0 [95% CI 1.2‐7.4]), and approval under exceptional circumstances or conditional approval (OR 2.7 [95% CI 1.1‐6.9]; for all *p* < 0.05) were associated with initiation of a registry. In the multivariate analysis, drugs considered as having an important level of innovation (OR 10.3 [95% CI 1.0‐103.9]) and orphan drugs (OR 2.8 [95% CI 1.0‐7.5]; both *p* < 0.05) remained significantly associated with registries. Therapeutic area, type of molecule, and size of safety population were not associated with a registry included in the marketing dossier.

## 4 DISCUSSION

Our study indicates that for one‐third of new drugs approved between 2007 and 2010, a commitment was made to perform studies in 1 or more registries to address remaining uncertainties of the drug's effects at the time of approval. The goal was primarily to collect further safety data (39 registries, 53%) or impact of drug use during pregnancy (27 registries, 37%), and only 7 registries (10%) collected data on both safety and drug effectiveness. Only for 9 out of 43 drugs, the registry was explicitly requested (imposed) by the CHMP as a specific obligation in the framework of the marketing authorization; the rest were proposed by the applicants. The majority of the registries involved were from existing disease registries (43 out of 73, 59%), implying that data collection was already ongoing and that a—sometimes only historical—control group may be available.

In a large proportion of new drug approvals, registries are planned for the post approval period, suggesting that regulators and/or companies feel a need to collect “real world” data to supplement incomplete knowledge at time of approval. This may not be a surprising development in an era of increasing availability of electronic health data.^15^, ^16^ The main reason for “real world” data collection is to address remaining safety concerns as well as generate data in low exposure groups notably pregnant women. This reflects the EU Pharmacovigilance legislation introduced in 2012. The legislation and the establishment of the Pharmacovigilance Risk Assessment Committee focus on all aspects of the risk management of drugs for human use,^17^ including the assessment of the risk of adverse reactions, while taking the therapeutic effect of the medicine into account. With regard to pregnancy data, in 2002 the FDA issued an amendment describing the requirement for the collection of pregnancy data through registries.^18^ A recent review concluded that these types of registries remain an important tool to collect safety data in the absence of randomized controlled trial data on pregnant women.^19^ Moreover, the FDA did accept registries for regulatory purposes in the evaluation of medical devices and is exploring further ways to use real‐world data in support of drug applications.^20^

Two‐thirds of the registries are existing disease registries, which is the approach promoted by EMA's Cross‐Committee Task Force on Registries. This is an initiative of the European regulators to facilitate better use of existing registries for the assessment of product safety and efficacy in daily clinical practice.^21^ An example of a disease registry set up and exclusively sponsored by one company are the following 2 orphan drugs. The first registry is for amifampridine which also includes patients who do not use amifampridine.^22^ The second registry is the Hunter Outcome Survey, in which patients with Hunter syndrome who are treated with enzyme replacement therapy are included.^23^ The majority of patients, however, received the drug marketed by the company. Recently, this approach was criticized by Hollak et al, who expressed a strong preference for disease registries to collect data, analyzed by independent statisticians, supervised by patients, healthcare professionals, and other relevant stakeholders, and to be launched early in the development of orphan drugs to obtain natural history data.^24^ We support this recommendation for a disease registry that is owned by an independent party. This guarantees that data of *all* drugs used can be included, thereby enabling future comparative analyses, which is in the interest of the patients and may be an instrument to control the price of drugs. Still, a third of all registries collect data on a single product; this limits their usability for continued learning.

Innovative drugs require more often a registry. These drugs fulfill unmet medical needs of patients eagerly awaiting these drugs. Innovative drugs are often “first‐in‐class” drugs with a new mechanism of action, where the full benefit‐risk profile—and in particular evidence about safety—may not be complete at the time of approval. Four out of 7 (57%) of the innovative drugs in this study were authorized through a conditional approval or an approval under exceptional circumstances, emphasizing that the data were limited at the time of marketing authorization. In addition, orphan drugs status by itself was an independent determinant for an approval with a registry in this study. This may be partly due to the large number of existing disease registries available in orphan diseases^25^ and is in line with our finding that in most cases data will be collected from existing disease registries. Earlier we have shown that higher levels of innovation or approval under exceptional circumstances/conditional approval are not related to more safety issues post approval.^5^, ^10^ Registry studies are considered valuable to increase our understanding of drug effects, especially for these drugs where the knowledge is incomplete at time of approval.

Our study has some limitations. The information about registries is retrieved from EPARs, published on the website of the EMA at the time of authorization. We used a more narrow definition of registries than described in the Good Vigilance Practices^3^; ie, “Any organised collection of data on patients all or not exposed to a specific drug may be considered a registry according to the Good Vigilance Practice (GVP) definitions.” We excluded 5 studies in electronic health records (secondary data analyses) and 7 open‐label short‐term (2 years or less) observational studies that could be considered to have met this wider definition. These studies were designed, however, for a specific research question rather than being intended for long‐term monitoring of patients in a registry with routine systematic collection of clinical data. These studies had not been acknowledged in the regulatory review as a registry study. One observational study proposed for roflumilast, which was not acknowledged as a registry in the regulatory review, could be considered as a registry according to our more narrow definition. In sensitivity analyses, the addition of this “registry” did not materially change our findings. Important innovations remained associated with a registry required at time of approval, although orphan drug status lost significance in the multivariate model (data not shown). Registries promised at a later stage in the drug life cycle might have been missed, and observational or effectiveness studies not designated as registries were not taken into account. These last sources may be less suitable for, eg, orphan drugs or for drugs exclusively used in a hospital setting. We observed that the rationale for the choice between the collection of data via a registry versus any other type of pharmacovigilance activity, such as post‐authorization safety studies or a retrospective cohort study in a database, is not clearly described in the EPAR. The rationale for a registry should follow from the benefit‐risk discussion of the drug, meaning that (1) it is indisputable which data are still needed to complete the understanding of the benefit‐risk profile of a drug and (2) these data can be retrieved from a registry during the post‐marketing phase.

Future studies should focus on the outcome of these planned registries in terms of studies actually undertaken post‐launch and the impact they may have had on the knowledge of the benefit‐risk ratio of a drug, eg, through changes in the drug labeling or through published findings in the literature. Challenges such as standardized protocols with clear objectives and endpoints, standards for data completeness, coding of data, the possibility to link register data to external data, and timelines for providing data are needed to share information between registry owners, companies, and regulators.^26^ Post‐approval studies in Europe and the USA to address safety and efficacy uncertainties at time of approval are, however, disappointing because recent reviews of such studies indicated that not many issues were resolved.^27^, ^28^ Reported delays in setting up imposed registries do not provide reassurance that these may provide timely information.^6^

Finally, we graded the innovation of a drug at time of approval. Clearly, the level of innovation may be subjective and time‐dependent. In a previous study, we have compared drugs classified using the Motola algorithm with some other classifications (Canadian Human Drug AdvisoryPanel and Prescrire International) and found a poor correlation.^10^ No system, however, can be considered as a “gold standard,”^10^ and we thus used 2 reviewers to grade drugs according to the transparently predefined criteria in the algorithm. Indeed, over time, with the registration of new drugs or alternative treatments becoming available, the value of drugs considered innovative at the time they were initially approved may diminish. Because in our study we looked for determinants of registries proposed at time of approval, such diminishing valuation of a drug after approval does not impact our study results.

We conclude that in one‐third of the newly approved drugs, a registry is required to provide additional data because of safety concerns. Most of these drugs were drugs with an important level of innovation and orphan drugs, for which there is high medical need. The majority of the registries involved are existing disease registries, implying that data collection is already ongoing and that a control group for comparison may be available.

## ETHICS STATEMENT

The authors state that no ethical approval was needed.

## CONFLICT OF INTEREST

The authors declare no conflict of interest.

## Supporting information

Appendix S1.
