# Supplementary material for: Registries supporting new drug applications
Source: Pharmacoepidemiol Drug Saf. 2017 Oct 6;26(12):1451–7. doi: 10.1002/pds.4332 (PMC5725674; doi:10.1002/pds.4332)
Supplement: Supplementary file 1 — Appendix S1. [file PDS-26-1451-s001.docx]

**Supplementary Appendix 1**

| **Active substance** | **Date of approval** | **Therapeutic Area (ATC-code)** | **Producttype** | **Level of innovation** | **Size of safety population** | **Orphan drug and approval** | **Primary goal for registry and if imposed** | **Drug or disease, incl. number of registries** |
| --- | --- | --- | --- | --- | --- | --- | --- | --- |
| abatacept | 21-may-07 | L04AA24 | biological | C: modest | 2778 | Regular | Safety & pregnancy | Disease  6 |
| amifampridine | 23-dec-09 | NO7 XX05 | small molecule | B: moderate | 282 | Orphan & Exceptional approval | Safety & effectiveness  Imposed | Disease  1 |
| aztreonam lysine | 21-sep-09 | J01DF01 | small molecule | B: moderate | 373 | Orphan & Conditional approval | Safety | Disease  1 |
| betaine anhydrous | 15-feb-07 | A16AA06 | small molecule | C: modest | 140 | Orphan & Regular | Safety | Drug  1 |
| canakinumab | 23-oct-09 | L04AC08 | biological | A: important | 104 | Exceptional approval | Safety & effectiveness  Imposed | Drug  1 |
| certolizumab pegol | 1-oct-09 | L04AB05 | biological | Pharm/Tech | 2367 | Regular | Safety | Disease  4 |
| darunavir | 2-feb-07 | J05AE10 | small molecule | A: important | 1783 | Conditional approval | Pregnancy | Disease  1 |
| denosumab | 26-mei-10 | M05BX04 | biological | C: modest | 13000 | Regular | Pregnancy | Disease  1 |
| eculizumab | 20-jun-07 | L04AA25 | biological | B: moderate | 716 | Orphan & Regular | Safety | Drug  1 |
| eltrombopag | 11-mar-10 | B02BX05 | small molecule | B: moderate | 422 | Orphan & Regular | Safety & pregnancy | Disease and drug  2 and 1 |
| eslicarbazepine acetate | 21-mai-09 | N03AF04 | small molecule | Pharm/Tech | 1694 | Regular | Pregnancy | Drug  1 |
| etravirine | 28-aug-08 | J05AG04 | small molecule | B: moderate | 1041 | Conditional approval | Pregnancy | Disease  1 |
| filgrastim (Zarzio) | 6-feb-09 | L03AA02 | biological | Pharm/Tech | 316 | Regular | Safety | Disease  1 |
| filgrastim (Ratiograstim) | 15-sep-08 | L03AA02 | biological | Pharm/Tech | 541 | Regular | Safety | Disease  1 |
| filgrastim (Nivestim)^1^ | 8-jun-10 | L03AA02 | biological | Pharm/Tech | 183 | Regular | Safety | Disease  2 |
| golimumab | 1-oct-09 | L04AB06 | biological | Pharm/Tech | 2758 | Regular | Safety & pregnancy | Disease  3 |
| human papillomavirus vaccine^1^ | 20-sep-07 | J07BM02 | vaccine | Pharm/Tech | 16142 | Regular | Pregnancy | Drug  2 |
| icatibant | 11-jul-08 | C01EB19 | small molecule | B: moderate | 962 | Orphan & Regular | Safety | Drug  1 |
| idursulfase | 8-jan-07 | A16AB09 | biological | B: moderate | 108 | Oprhan & Exceptional approval | Safety & effectiveness  Imposed | Disease  1 |
| influenza virus surface antigens | 2-mai-07 | J07BB02 | vaccine | Pharm/Tech | 646 | Exceptional approval | Pregnancy | Drug  1 |
| lacosamide^1^ | 29-aug-08 | NO3AX18 | small molecule | Pharm/Tech | 1338 | Regular | Pregnancy | Disease  2 |
| lenalidomide^1^ | 14-jun-07 | L04AX04 | small molecule | B: moderate | 353 | Orphan & Regular | Safety  Imposed | Drug  1 |
| maraviroc | 18-sep-07 | J05AX09 | small molecule | A: important | 840 | Regular | Safety & pregnancy | Disease and drug  1 and 1 |
| mecasermin^1^ | 3-aug-07 | H01AC03 | small molecule | B: moderate | 1516 | Oprhan & Exceptional approval | Safety  Imposed | Drug  1 |
| meningococcal group A, C, W-135 and Y conjugate vaccine | 15-mar-10 | J07AH08 | vaccine | Pharm/Tech | 6745 | Regular | Pregnancy | Disease  1 |
| nicotinicacid / laropiprant^2^ | 3-jul-08 | C10AD52 | small molecule | Pharm/Tech | 2552 | Regular | Pregnancy | Drug  1 |
| pandemic influenza vaccine (h1n1) (split virion, inactivated, adjuvanted)^3^ | 23-mar-10 | J07BB02 | vaccine | Pharm/Tech | 3456 | Conditional approval | Pregnancy  Imposed | Drug  1 |
| pandemic influenza vaccine (h1n1) (split virion, inactivated, adjuvanted)^3^ | 8-jun-10 | J07BB02 | vaccine | Pharm/Tech | 1020 | Conditional approval | Pregnancy  Imposed | Drug  1 |
| plerixafor | 31-jul-09 | L03AX16 | small molecule | C: modest | 1161 | Orphan & Regular | Safety | Disease  1 |
| prasugrel^1^ | 25-feb-09 | B01AC22 | small molecule | Pharm/Tech | 8656 | Regular | Safety | Disease  3 |
| prepandemic influenza vaccine (h5n1) (surface antigen, inactivated, adjuvanted) | 29-nov-10 | J07BB02 | vaccine | Pharm/Tech | 3983 | Regular | Pregnancy | Disease  1 |
| raltegravir | 20-dec-07 | J05AX08 | small molecule | A: important | 899 | Conditional approval | Pregnancy | Disease  1 |
| rilonacept^1,3^ | 23-oct-09 | L04AC04 | biological | A: important | 614 | Orphan & Exceptional approval | Safety & efficacy  Imposed | Drug  2 |
| romiplostim | 4-feb-09 | B02BX04 | biological | C: modest | 271 | Orphan & Regular | Safety & pregnancy | Drug  6 |
| rufinamide | 16-jan-07 | N03AF03 | small molecule | A: important | 1978 | Orphan & Regular | Safety& pregnancy | Disease  2 |
| sapropterin | 2-dec-08 | A16AX07 | small molecule | B: moderate | 647 | Orphan & Regular | Safety & effectiveness | Drug  1 |
| telbivudine | 24-apr-07 | J05AF11 | small molecule | B: moderate | 1491 | Regular | Pregnancy | Disease  1 |
| tocilizumab | 16-jan-09 | L04AC07 | biological | C: modest | 2439 | Regular | Safety & Pregnancy | Disease  4 |
| tocofersolan | 24-jul-09 | A11HA08 | small molecule | B: moderate | 167 | Exceptional approval | Safety  Imposed | Drug  1 |
| ulipristal | 15-mai-09 | G03AD02 | small molecule | Pharm/Tech | 3560 | Regular | Pregnancy | Drug  1 |
| ustekinumab | 16-jan-09 | L04AC05 | biological | B: moderate | 2266 | Regular | Safety & pregnancy | Disease  3 |
| velaglucerase alfa | 26-aug-10 | A16AB10 | biological | Pharm/Tech | 94 | Orphan & Regular | Safety & effectiveness | Disease  1 |
| vernakalant hydrochloride | 1-sep-10 | C01BG11 | small molecule | Pharm/Tech | 883 | Regular | Safety | Drug  1 |

1. Details were obtained from the Risk Management Plans (RMP) and study reports, retrieved from the database available at the Medicine Evaluation Board (MEB).
2. The company decided to voluntarily withdraw the marketing authorisations. This followed the CHMP recommendation to suspend the marketing authorisations of this product.
3. The company decided to voluntarily withdraw the marketing authorisation for these products for commercial reasons. The products had never been placed on the market in any country of the European Community.
